# Are health promoting lifestyles associated with pain intensity and menstrual distress among Iranian adolescent girls?

**DOI:** 10.1186/s12887-022-03639-x

**Published:** 2022-10-05

**Authors:** Elahe Cholbeigi, Shaghayegh Rezaienik, Narges Safari, Kerrie Lissack, Mark D Griffiths, Zainab Alimoradi

**Affiliations:** 1 Students research committee, Qazvin University of Medical Sciences, Qazvin, Iran; 2 Graduate School of Education, University of Exeter, Exeter, UK; 3 International Gaming Research Unit, Psychology Department, Nottingham Trent University, Nottingham, UK; 4 Social Determinants of Health Research Center, Research Institute for Prevention of Non- Communicable Diseases, Qazvin University of Medical Sciences, Qazvin, Iran

**Keywords:** Health-promoting lifestyle, Dysmenorrhea, Menstrual distress, Adolescents

## Abstract

**Objective:**

The present study investigated the relationship between a health-promoting lifestyle and menstrual pain intensity and distress among adolescent girls in Qazvin.

**Methods:**

A cross-sectional survey study using a two-stage sampling method was conducted. The participants (n = 986) were female high school students aged 14–19 years living in Qazvin. Random cluster sampling was used to recruit participants from schools and classes from each grade. An online survey was provided to all participants to complete. Data were collected using a Demographic and Menstrual Characteristics Checklist, the Visual Analogue Scale (VAS) to assess dysmenorrhea intensity, the Andresh Milsom Scale (AMS) to assess dysmenorrhea severity, the Moos Menstrual Distress Questionnaire (MMDS) to assess menstrual distress, and the Health-Promoting Lifestyle Profile (HPLP) to assess a health promoting lifestyle. Data were analyzed using an univariable linear regression model at a significance level of 0.05.

**Results:**

The findings indicated that 421 participants (42.7%) experienced dysmenorrhea with a mean duration of 2.24 days (SD = 1.57) and a mean pain intensity of 4.62 on the VAS (SD = 2.87). The mean score on the menstrual distress on the MDDS was 13.55 (SD = 8.88) and the mean score on the HPLP was 2.55 (SD = 0.50). Based on the results of univariable linear regression, nutrition (β=-0.18, *p* < 0.001) and exercise (β=-0.17, *p* < 0.001) had the most significant effect on the severity of dysmenorrhea pain. Also, self-actualization (β=-0.29, *p* < 0.001), stress management (β=-0.25, *p* < 0.001) and nutrition (β=-0.25, *p* < 0.001) had the most significant effect on menstrual distress.

**Conclusion:**

Based on these findings, it is suggested that (i) improving nutrition and exercise might reduce the severity of dysmenorrhea pain and (ii) improving self-actualization, stress management and nutrition might reduce the severity of menstrual distress. Overall, it seems that improving health-promoting lifestyle behaviors can help improve the severity of dysmenorrhea pain and menstrual distress among adolescent girls.

## Introduction

Dysmenorrhea (i.e., painful menstruation) is one of the most common reproductive problems and occurs as acute pelvic pain around the time of menstruation [1]. It is defined as primary or secondary according to the underlying pathological condition. Primary dysmenorrhea is the presence of painful menstruation in the absence of provable pelvic disease and secondary dysmenorrhea is the occurrence of bleeding and painful menstruation due to pathological pelvic problems [2]. Primary dysmenorrhea usually begins one to two years after menarche and with the onset of ovulation [1, 3]. Different prevalence rates ranging from 16% to 91% have been reported for primary dysmenorrhea [4–6], with moderate to severe pain in 40% [7]. Adolescents and young adults often experience dysmenorrhea without any underlying cause (primary dysmenorrhea) [1].

Dysmenorrhea is usually accompanied by menstrual distress. Menstrual distress can comprise both physical and psychological symptoms including irritability and tenderness of the breasts, back pain, skin disorders, fatigue, palpitations, withdrawal, nausea and vomiting, abdominal pain, and general weakness [8]. It can occur before, during and/or after menstruation [9]. Menstrual distress, like dysmenorrhea, is a common problem for women of childbearing age [10] with prevalence rates reported to be 75%–94% [11].

Dysmenorrhea, especially when accompanied by symptoms of menstrual distress, is a major factor in disrupting the quality of life and social activities of young women [12, 13]. Although primary dysmenorrhea is not life-threatening, it can adversely affect performance and quality of life among females [14]. Dysmenorrhea has many adverse effects on women’s lives, including reduced daily activities, lower academic performance in adulthood, decreased sleep quality, and negative effects on mood (e.g., anxiety and depression) [15]. With the increase in medical expenses and medical care and the decrease in the efficiency of individuals, dysmenorrhea has always been considered in terms of economic and social effects [5].

Primary dysmenorrhea occurs due to increased or unbalanced production of prostaglandins which leads to increased uterine contractions with a dysrhythmic pattern, increased basal tone, and increased active pressure. Increased uterine contraction activity, decreased uterine blood flow, and increased peripheral nerve sensitivity are involved in causing pain [16]. Several factors including age, history of marriage, body mass index, family history of dysmenorrhea, and low economic and social status can affect the severity of primary dysmenorrhea [2, 5, 12, 13, 17, 18]. Also, psychological factors such as anxiety, depression, personality traits, high emotional disorder, and psychological symptoms [19–21] can affect the severity of primary dysmenorrhea. In addition, nutrition and exercise are among other factors that have been considered in relation to the severity of dysmenorrhea [22, 23].

By reviewing the factors affecting the severity of dysmenorrhea (nutrition, exercise, stress, mental health, etc.) it appears that individuals’ lifestyles can affect the severity of dysmenorrhea. Lifestyle is a normal daily activity that individuals have accepted in their lives and these activities affect individuals’ health [24]. It has been proposed that health-promoting lifestyles comprise six dimensions (i.e., self-actualization, health responsibility, exercise, nutrition, interpersonal support, and stress management) [25]. Few studies have examined the relationship between these lifestyle-related factors and dysmenorrhea, and the results have not been consistent. For example, Kazama et al. reported the possible relationship between lifestyle factors and dysmenorrhea [26]. Baville et al. reported a significant univariable difference in nutrition, exercise, stress, and social relationships among those with dysmenorrhea (compared to those without it) but the difference was no longer significant following multivariable regression analysis [27]. Naghizadeh et al. reported significant differences between those with and without dysmenorrhea regarding health promoting lifestyles concerning aspects of exercise, stress management, and self-actualization [28].

Unlike dysmenorrhea, the lifestyle factors affecting menstrual distress have not received enough research attention. Considering the variety of different factors affecting the severity of dysmenorrhea and menstrual distress, it appears that examining this issue from a general perspective and focusing on the relationship between health-promoting behaviors and the severity of dysmenorrhea and menstrual distress (as prevalent problems of reproductive age women) can be a guide for designing and implementing lifestyle-based interventions to improve dysmenorrhea. Therefore, present study investigated the association between a health-promoting lifestyle and menstrual pain intensity and distress among adolescent girls aged 14 to 19 in Qazvin (Iran).

## Methods

### Design and participants

A cross-sectional study was conducted from January to April 2021 with the participation of 986 Iranian female high school students in Qazvin. The inclusion criteria were being enrolled in the second year of high school, being in the age range of 14–19 years, and the onset of menstruation being at least two years. The exclusion criteria were a history of (self-reported) physical or mental illness such as heart disease, diabetes, hypertension, hyperlipidemia, etc., and unwillingness to participate in the study.

### Sampling procedure

Sampling was performed via two-stage random sampling method. In the first stage, random cluster sampling was performed. Qazvin city has two educational districts with a total of 20 female specific public, non-governmental, and vocational technical schools for secondary high school education (Grades 10, 11, and 12). On average, each school has three classes for each grade. Random cluster sampling was performed to select three schools as the main clusters in each educational district (totaling six schools). Following this, using second-stage random cluster sampling, one class was randomly selected from each grade (totaling three class from each school). In selected classes, all students were invited to complete the study survey. Data were collected using an online survey hosted on the *Porsline* platform. The link to the online survey was sent to potential participants via social media apps (*WhatsApp* and *Telegram*), and SMS to the participants.

### Sample size estimation

Considering the minimum prevalence of 50% dysmenorrhea among students based on a previous Iranian meta-analysis [6], α error of 0.01 and *d* error of 0.05, the required sample size was calculated based on the following formula resulting in a sample size of 660 individuals.

$$n=\frac{{\left({z}_{1-\frac{\propto }{2}}\right)}^{2}\text{*}p\text{*}(1-p)}{{d}^{2}}=\frac{\left({2.57}^{2}\right)\text{*}0.5\text{*}0.5}{{0.05}^{2}}=660$$

### Measures

Data were collected using a Demographic and Menstrual Information Checklist, the Visual Analogue Scale, the Andresh Milsom Scale, the Moos Menstrual Distress Questionnaire, and the Health Promoting Lifestyle Profile.

*Demographic and Menstrual Information Checklist (DMIC)*: The DMIC was used to collect information concerning demographic and fertility characteristics including age, menarche age, level of education, field of study, relationship status, characteristics of the menstrual cycle (length of menstrual period, length of bleeding period), history of analgesic use and its amount, drug addiction, height, weight, body mass index, history of dysmenorrhea or menstrual distress in mother and sister, history of secondary dysmenorrhea, and underlying diseases. The checklist was compiled based on the objectives of the study. Its validity was checked using qualitative content validity and the opinion of the academic members affiliated to Midwifery and Obstetric departments of Qazvin University of Medical Sciences.

*Visual analogue scale (VAS)*: The VAS was used to assess the intensity of menstrual pain. The VAS comprises a 10 cm line that has zero at one end indicating *“complete painlessness”* and 10 at the other end indicating the *“most severe pain imaginable”* [29]. Due to its simplicity and ease of use, the VAS is frequently used in study of pain [30]. The validity and reliability of this tool have been confirmed to evaluate the severity of dysmenorrhea pain [31].

*Andresh Milsom Scale (AMS)*: The AMS was used to assess the severity of menstrual pain. In this scale, dysmenorrhea pain comprises four subgroups: no pain, mild pain without the need for painkillers, moderate pain that can be relieved by taking painkillers, and severe pain that does not go away with painkillers [32]. The validity and reliability of this scale had been assessed and confirmed [32].

*Moos Menstrual Distress Questionnaire (MMDQ)*: The 47-item MMDQ was used to assess the effect of menstrual distress on a person’s daily activities. The MMDQ examines individuals’ general complaints concerning various symptoms during menstruation on a four-point Likert scale from 1 (asymptomatic) to 4 (the most severe state). The validity and reliability of the Persian version of the short form of this questionnaire has been psychometrically evaluated and confirmed as being good [33]. In the present study, the internal reliability of the scale was very good (Cronbach’s alpha = 0.88).

*Health-Promoting Lifestyle Profile (HPLP)*: The 52-item HPLP was used to assess health-promoting lifestyle. The HPLP comprises six dimensions including self-actualization, health responsibility, exercise, nutrition, interpersonal support, and stress management. Items are rated on a four-point scale (never = 1, sometimes = 2, often = 3, permanent = 4). To calculate the score in each domain, the average is calculated and therefore the score of each domain varies between 1 and 4 [25]. The validity and reliability of the Persian version of the HPLP among Iranian adolescents has been psychometrically evaluated and confirmed as being good [34]. In the present study, the internal reliability of the scale was excellent (Cronbach’s alpha = 0.95).

### Statistical analysis

SPSS statistical software version 25 was used for data analysis. Descriptive and inferential statistical methods including univariable linear regression model were used to investigate the relationship between dysmenorrhea pain intensity and menstrual distress with health promoting lifestyle dimensions. In all regression models, the total score of dysmenorrhea pain intensity and menstrual distress was entered as dependent variable and the HPLP total and subscale scores were entered as independent variables. Assumptions of linear regression method including normal distribution of dependent variables (dysmenorrhea pain intensity and menstrual distress), and lack of outlier data were controlled before running the models. The significance level in the present study was *p* < 0.05.

### Ethical considerations

The proposal of the present study was reviewed and approved by the Research Council of Qazvin University of Medical Sciences and the Ethics Committee in Biological Research of Qazvin University of Medical Sciences (IR.QUMS.REC.1398.187). The necessary permits for conducting the study were obtained from the Research Vice-Chancellor at the Ministry of Education as the guardian of the students. Moreover, informed consent to participate in the study was obtained from all teenage participants and their parents, after expressing objectives, and assuring the participants about the confidentiality of their data and their right to withdraw from the study at any time.

## Results

As aforementioned, 968 adolescent girls participated in the study. The mean age of participants was 16.19 years (SD = 1.22), body mass was 21.62 (SD = 3.96), and the mean onset of menstruation was 12.72 years (SD = 1.25). Approximately two-fifths of participants stated that they had dysmenorrhea (n = 421; 42.7%). The mean duration of menstrual pain was 2.24 days (SD = 1.57) and the mean intensity of menstrual pain on the VAS was 4.62 (SD = 2.87). Based on AMS menstrual severity scores, 454 participants had moderate dysmenorrhea (46%). The mean score of menstrual distress on the MMDS was 13.55 (SD = 8.88) and 484 participants had a family history of dysmenorrhea (49.1%). Table 1 presents the demographic and menstrual characteristics of the participants.

**Table 1:** Distribution of participants’ characteristics (N = 986)

	Range	Mean (SD)
Age (year)	14–19	16.19 (1.22)
BMI (kg/m^2^)	13.52–38.28	21.62 (3.96)
Menarche age (year)	7–17	12.72 (1.25)
Menstrual interval (days)	20–90	27.85 (5.35)
Menstrual duration (days)	2–10	5.86 (1.54)
Menstrual distress	0–45	13.55 (8.88)
Dysmenorrhea pain intensity based on VAS score	0–9	4.62 (2.87)
		No (%)
Intensity of pain based on Andresh Milson Scale score	No pain	85 (8.6)
	Mild pain	333 (33.8)
	Moderate pain	454 (46.0)
	Severe pain	114 (11.6)
Having regular menses	No	411 (41.7)
	Yes	575 (58.3)
Pain relief use	No	432 (43.8)
	Yes	554 (56.2)
Family history dysmenorrhea	No	502 (50.9)
	Yes	484 (49.1)
History of chronic disease	No	943 (95.6)
	Yes	43 (4.4)

The mean score of health-promoting lifestyle on the HPLP among participants was 2.55 (SD = 0.50). The highest score was on self-actualization (3.03; SD = 0.65) and the lowest score was on health responsibility (2.12; SD = 0.64). Based on the results of univariable linear regression (Table 2), there was a significant relationship between the total score and dimensions of health promoting lifestyle with the severity of dysmenorrhea pain as well as menstrual distress. Among the dimensions of health-promoting lifestyle profile, nutrition (β=-0.18, *p* < 0.001) and exercise (β=-0.17, *p* < 0.001) had the most significant effect on the severity of dysmenorrhea. Therefore, the severity of dysmenorrhea pain was significantly lower among participants with higher scores of nutrition and exercise. The results of regression model in the relationship between health-promoting lifestyle dimensions and menstrual distress showed that self-actualization (β=-0.29, *p* < 0.001), stress management (β=-0.25, *p* < 0.001) and nutrition (β=-0.25, *p* < 0.001) had the most significant effect on menstrual distress. Therefore, the severity of menstrual distress was lower among participants with higher scores of self-actualization, stress management, and nutrition. Overall, health promoting life style dimensions had a stronger effect on menstrual distress compared to dysmenorrhea pain intensity.

**Table 2:** Results of univariable regression analysis assessing the association of dysmenorrhea pain intensity and menstrual distress with HPLP dimensions

	Mean (SD)	Dysmenorrhea pain intensity					Menstrual distress				
		B (95% CI)	S.E.	β	***p***-value	Adj R^2^	B (95% CI)	S.E.	β	***p***-value	Adj R^2^
Health responsibility	2.12 (0.64)	-0.59 (-0.87; -0.31)	0.14	-0.13	**< 0.001**	0.02	-2.66 (-1.80; -3.51)	0.44	-0.19	**< 0.001**	0.04
Exercise	2.24 (0.70)	-0.68 (-0.93; -0.42)	0.13	-0.17	**< 0.001**	0.03	-2.88 (-2.11; -3.66)	0.40	-0.23	**< 0.001**	0.05
Nutrition	2.59 (0.55)	-0.93 (-1.25; -0.61)	0.16	-0.18	**< 0.001**	0.03	-3.96 (-2.98; -4.94)	0.50	-0.25	**< 0.001**	0.06
Self-actualization	3.03 (0.65)	-0.63 (-0.90; -0.35)	0.14	-0.14	**< 0.001**	0.02	-3.95 (-3.13; -4.78)	0.42	-0.29	**< 0.001**	0.08
Interpersonal support	2.69 (0.56)	-0.47 (-0.79; -0.15)	0.16	-0.09	**< 0.001**	0.01	-3.07 (-2.09; -4.05)	0.50	-0.19	**< 0.001**	0.04
Stress management	2.61 (0.60)	-0.62 (-0.92; -0.33)	0.15	-0.13	**< 0.001**	0.02	-3.70 (-2.81; -4.59	0.45	-0.25	**< 0.001**	0.06
HPLP (total score)	2.55 (0.50)	-0.99 (-1.35; -0.64)	0.18	-0.17	**< 0.001**	0.03	-5.10 (-6.17; -4.04)	0.54	-0.29	**< 0.001**	0.08
N.B. Bold type refers to significant results											

## Discussion

Findings from the present study showed that higher scores on the nutrition and exercise dimensions on the Health-Promoting Lifestyle Profile (HPLP) were significantly associated with lower severity of dysmenorrhea pain. The findings of the study are consistent with previous studies in that the modification of eating behaviors [35–37] and exercise [37–40] were associated with a reduction in the severity of dysmenorrhea. A possible relationship between lifestyle factors and dysmenorrhea was reported by Kazama et al. [26]. Baville et al. also reported a significant difference in nutrition, exercise, stress, and social relationships among those with dysmenorrhea compared to those without [27]. Naghizadeh et al. reported significant differences between those with dysmenorrhea (compared to those without) regarding health promoting lifestyle aspects (i.e., exercise, stress management, and self-actualization) [28]. Consistently, nutrition and exercise are among the most influential life style behaviors influencing the experience and severity of dysmenorrhea.

The results of systematic review of Armor et al. also showed that among lifestyle-related interventions, exercise had the highest effect in reducing the severity of menstrual pain [39]. Exercise releases endorphins, as well as promoting relaxation and stress reduction which can reduce the severity and duration of dysmenorrhea [41]. The results of another recent systematic review [22] reported an association between some nutritional factors and primary dysmenorrhea. More specifically, increasing the consumption of fruits and vegetables as sources of many vitamins and minerals, fish, and milk (and other dairy products) was positively associated with less menstrual pain [22]. There was also a negative association between dysmenorrhea and skipping meals and following a diet for weight loss. Therefore, attention should be paid to improving women’s eating behaviors to have an adequate and balanced diet [22].

The results of the present study showed that 42.7% of participants reported having dysmenorrhea, and that for 46% of them the pain severity was moderate. Although primary dysmenorrhea is one of the most common problems of reproductive age, differing prevalence rates have been reported in the extant literature. In a systematic review of Iranian studies, the prevalence of primary dysmenorrhea was reported to be 71% overall and almost 59% among adolescents [36]. This is consistent with present study. The results of a review of 15 studies between 2002 and 2011 reported a prevalence of primary dysmenorrhea of ​​16%–91% among women of childbearing age, with severe dysmenorrhea ranging from 2% to 29% [5]. French et al. (2005) reported prevalence rates of dysmenorrhea among adolescent girls ranging between 20% and 90% [4]. Parker reported a prevalence rate 93% for dysmenorrhea among 1,803 high school seniors, with 40% having moderate to severe pain [7]. The difference in prevalence rates of dysmenorrhea might be due to the (i) use of different scales to assess the experience and intensity of dysmenorrhea, (ii) different age and reproductive characteristics of participants, and (iii) different study designs.

The results of regression model regarding association of health-promoting lifestyle dimensions with menstrual distress showed that self-actualization, stress management, and nutrition had the most significant effects, respectively. Unlike dysmenorrhea, the predictive role of lifestyle behaviors in menstrual distress have rarely been investigated. A similar finding was observed by Noh et al. who reported a significant negative relationship between lifestyle and menstrual distress [42]. Yamamoto et al. also reported a relationship between stress and premenstrual syndrome symptoms and menstrual problems [43]. Yang reported that by using yoga intervention as a psychological approach which can control an individual’s stress, the severity of menstrual distress was significantly reduced [44]. Therefore, it seems that to improve the symptoms of menstrual distress, which includes physical and psychological dimensions, attention should be paid to promoting both physical and psychological dimensions of a health-promoting lifestyle.

### Strength and limitations

To best of the authors’ knowledge, the present study is one of the first studies to assess the association of a health-promoting lifestyle with intensity of dysmenorrhea and menstrual distress. Using the HPLP measure provided the opportunity to assess association of different aspects of health-promoting lifestyle with two common problems among adolescents. Having an appropriate sample size selected via random sampling method enhanced the generalizability of results for female adolescents experiencing dysmenorrhea or menstrual distress. In addition to these study strengths, the findings should be interpreted considering the study’s limitations. The cross-sectional nature of the study was unable identify any causal relationships between health-promoting behaviors and the severity of dysmenorrhea pain or menstrual distress. In addition, to examine the variables in the present study, psychometric scales reliant on self-report were used which did not have an objective assessment of nutritional status and exercise. Data were collected from adolescents living in urban area and going to secondary high schools. Therefore, generalization of the results to adolescents living in rural arrears and to those not attending high schools is another limitation.

## Conclusion

According to the study’s results, nutrition and exercise are associated with the severity of dysmenorrhea pain. Additionally, self-actualization, stress management, and nutrition are associated with the severity of menstrual distress. Therefore, it seems that interventions considering health promoting lifestyle can be helpful in managing dysmenorrhea and menstrual distress among adolescent girls. In this regard, further studies can be designed to assess the efficacy of improving health-promoting lifestyle behaviors (via different educational method) on severity of dysmenorrhea pain and menstrual distress among adolescent girls.

## Data Availability

Data and materials will be provided upon email to corresponding author.

## Abbreviations

AMS: Andresh Milsom Scale.
HPLP: Health-Promoting Lifestyle Profile.
MMDQ: Moos Menstrual Distress Questionnaire.
SD: Standard Deviation.
VAS: Visual Analogue Scale.
